# Circulating *VEGF* and *eNOS* variations as predictors of outcome in metastatic colorectal cancer patients receiving bevacizumab

**DOI:** 10.1038/s41598-017-01420-0

**Published:** 2017-05-02

**Authors:** Giorgia Marisi, Emanuela Scarpi, Alessandro Passardi, Oriana Nanni, Angela Ragazzini, Martina Valgiusti, Andrea Casadei Gardini, Luca Maria Neri, Giovanni Luca Frassineti, Dino Amadori, Paola Ulivi

**Affiliations:** 1 Biosciences Laboratory, Istituto Scientifico Romagnolo per lo Studio e Cura dei Tumori (IRST) IRCCS, via P. Maroncelli, 40, 47014 Meldola, Italy; 2 Unit of Biostatistics and Clinical Trials, Istituto Scientifico Romagnolo per lo Studio e Cura dei Tumori (IRST) IRCCS, via P. Maroncelli, 40, 47014 Meldola, Italy; 3 Department of Medical Oncology, Istituto Scientifico Romagnolo per lo Studio e Cura dei Tumori (IRST) IRCCS, via P. Maroncelli, 40, 47014 Meldola, Italy; 4 Department of Morphology, Surgery and Experimental Medicine, University of Ferrara, via Fossato di Mortara 70, 44100 Ferrara, Italy

## Abstract

Novel predictive biomarkers are needed to improve patient selection and optimize the use of bevacizumab (B) in metastatic colorectal cancer. We analyzed the potential of five circulating biomarkers to predict B efficacy and monitor response. Peripheral blood samples collected at baseline, at the first clinical evaluation and at progression were available for 129 patients enrolled in the prospective multicentric *ITACa* trial and randomized to receive FOLFOX4/FOLFIRI (CT) with (64 patients) or without B (65 patients). VEGF-A, eNOS, EPHB4, COX2 and HIF-1α mRNA levels were measured by qRT-PCR. Baseline marker expression levels and their modulation during therapy were analyzed in relation to objective response, progression-free survival and overall survival (OS). VEGF and eNOS expression was significantly correlated in both groups (Spearman’s correlation coefficient = 0.80; P < 0.0001 and 0.75; P < 0.0001, respectively). B-treated patients with >30% reduction in eNOS and VEGF levels from baseline to the first clinical evaluation showed better OS than the others (median OS 31.6 months, 95% CI 21.3–49.5 months and median OS 14.4 months, 95% CI 9.0–22.7 months, respectively, HR 0.38, 95% CI 0.19–0.78, P = 0.008). A reduction in eNOS and VEGF expression from baseline to the first clinical evaluation may indicate a response to B.

## Introduction

Bevacizumab (B), a humanized monoclonal antibody targeting vascular endothelial growth factor (VEGF-A), has proven clinical efficacy when used in first- or second-line treatment in association with fluorouracil-based chemotherapy (CT) in metastatic colorectal cancer patients (mCRC)^1–3^. To date there are no predictive biomarkers capable of identifying patients who are most likely to benefit from this treatment^4^. Plasma or serum concentrations of baseline VEGF-A have been analyzed in relation to drug efficacy, but with contrasting results^5–8^. Pretreatment total circulating VEGF-A seems to be prognostic for outcome in mCRC patients, but a predictive role for B efficacy has yet to be demonstrated^5, 9^. An increase in serum VEGF-A concentration after an initial decrease has been proposed as a predictive marker of poor response and of reactive resistance to chemotherapy plus B^10^.

We previously described the role of endothelial nitric oxide synthase (eNOS) polymorphisms as possible predictive biomarkers of B efficacy^11^ in patients enrolled in the *ITACa* (Italian Trial in Advanced Colorectal Cancer) trial, a prospective randomized phase III multicentric study designed to investigate the role of B treatment in mCRC patients^12^. In particular, patients carrying a specific haplotype combination of 2 *eNOS* polymorphisms (*eNOS* + 894 G/T and *eNOS* VNTR 4a/b) showed significantly longer progression-free survival (PFS) and overall survival (OS) and a higher overall response rate (ORR) than those with other genotypes^11^.

*eNOS* is a constitutively expressed gene in the endothelium involved in the production of nitric oxide (NO), which plays a central role in maintaining endothelial cell functional integrity, regulating hemodynamics, and establishing collateral circulation^13, 14^.

The expression of other biomarkers seems to be correlated with B response. Patients with low ephrin type-B receptor 4 (EPHB4) mRNA levels in tumor tissue have a higher response to B than those with high levels^15^. EPHB4 belongs to a large family of receptor tyrosine kinases and mediates arteriovenous differentiation during embryonic development, regulating induction and maturation of newly forming vessels in the adult in both physiological and pathological conditions^15–17^.

Other factors are hypothesized to play a role in determining B sensitivity or resistance. Cyclooxygenase-2 (COX2) is a key enzyme for inflammatory cytokine-induced angiogenesis^18^ whose expression levels may consequently influence B activity. Moreover, as hypoxia represents an important event during anti-angiogenic therapy, hypoxia inducible factor 1 alpha (HIF-1α) may represent an important prognostic factor during B treatment.

We measured the blood circulating mRNA expression of VEGF-A, eNOS, EPHB4, COX2 and HIF-1α to study the predictive role of these markers at baseline and to monitor B efficacy during treatment.

## Results

### Patient characteristics

The clinical-pathologic characteristics of patients enrolled in the CT + B or CT groups are shown in Table 1. Median age was 69 (range 34–83) and 67 years (range 33–82), respectively. Baseline patient characteristics were well balanced between groups. One hundred patients had liver metastases (35 had liver metastases only and 65 also had extra-hepatic lesions). The remaining 29 patients had only extra-hepatic lesions. Median PFS and OS of CT + B patients were 9.6 months (95% confidence intervals [CI], 8.3–12.4) and 21.4 months (95% CI, 13.9–28.8), respectively, while median PFS and OS of CT patients were 9.1 months (95% CI, 8.3–10.4) and 24 months (95% CI, 18.5–28.0), respectively. With regard to tumor localization, 50 patients had a right-side tumor and 76 a left-side tumor. Within the right-side group, PFS was significantly higher in CT + B patients (12.6 months [95% CI, 8.6–16.0]) compared to CT patients (9.0 months [95% CI, 5.1–10.3] (P = 0.020). The difference was evident but not significant with regard to OS (27.5 months [95% CI, 15.9–35.7] for CT + B *vs*. 20.3 months [95% CI, 12.1–24.5] for CT) (P = 0.173). No differences were seen in the group of patients with a left-side tumor. Median PFS and OS of the entire population were 9.3 months (95% CI, 8.9–10.4) and 22.7 months (95% CI, 18.8–27.1), respectively. Median follow-up was 52 months (range 1–77).

**Table 1:** Patient characteristics.

Patient characteristics	CT + B (N = 64) No. (%)	CT (N = 65) No. (%)
Median age, years (range)	69 (34–83)	67 (33–82)
Gender		
Male	40 (62.5)	35 (53.8)
Female	24 (37.5)	30 (46.2)
Performance Status (ECOG)		
0	54 (84.4)	53 (81.5)
1−2	10 (15.6)	12 (18.5)
Tumor localization		
Rectum	20 (31.2)	19 (29.2)
Colon	44 (68.8)	46 (70.8)
Right side	26^#^ (41%)	24^#^ (38%)
Left side	37^#^ (59%)	39^#^ (62%)
Stage at diagnosis		
I–III	14 (21.9)	15 (23.1)
IV	50 (78.1)	50 (76.9)
Grade		
1 + 2	32 (59.3)	34 (60.7)
3	22 (40.7)	22 (39.3)
Unknown/missing	10	9
CT regimen		
FOLFOX4	39 (60.9)	38 (58.5)
FOLFIRI	25 (39.1)	27 (41.5)
*KRAS* status*		
Wild type	37 (59.7)	36 (60.0)
Mutated	25 (40.3)	24 (40.0)
Unknown/missing	2	5
Prior cancer therapy		
Surgery	51 (79.7)	48 (73.8)
Radiotherapy	6 (9.4)	6 (9.2)
Adjuvant chemotherapy	10 (15.6)	9 (13.8)

CT, chemotherapy; B, bevacizumab.

*Required by amendment no. 1 of 3^rd^ May 2009; ^#^Information about which side of the colon was involved was not available for one patient in the CT + B group and 2 patients in the CT group.

### Baseline circulating levels of VEGF, eNOS, EPHB4, COX2 and HIF-1α with respect to patient outcome

No significant correlation was found between the main clinical-pathologic characteristics of patients and median baseline biomarker levels (Supplementary Table S1). There were 37 responders and 25 non responders in the CT + B arm, and 38 responders and 27 non responders in the CT group. No statistically significant differences were seen between the median baseline biomarker levels of responders and non responders (Supplementary Table S2). Furthermore, there were no substantial differences observed between baseline values and PFS and OS (Supplementary Table S3).

### Circulating biomarker variations during treatment in relation to treatment response

We analyzed the variation in circulating levels of the 5 biomarkers from baseline to the first clinical evaluation. Within the B group, patients with >30% reduction in EPHB4 levels showed a higher rate of response (complete or partial response) than those with no variation. In particular, 24 (75%) of the 32 responders had >30% reduction in EPHB4 levels compared to 9 out of 19 (47%) non-responders (P = 0.048). We also observed a trend towards significance in the control group (P = 0.064). No substantial differences were seen between other biomarkers and response.

### Circulating biomarker variations during treatment in relation to patient survival

**Figure 1:**
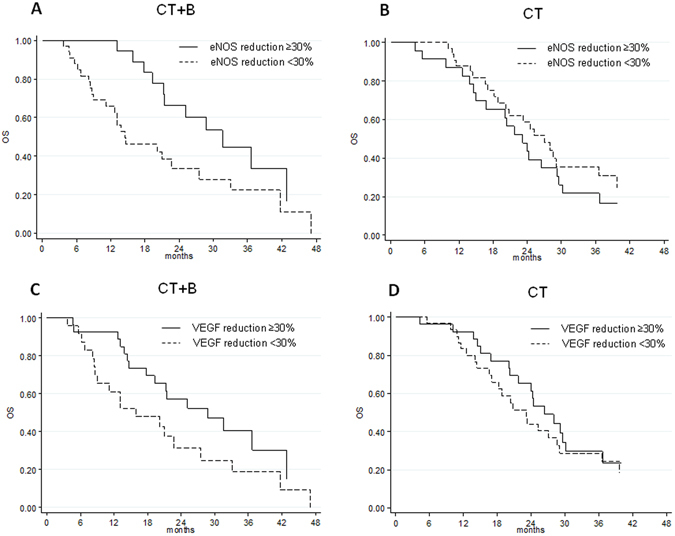
Patient flow diagram.

We evaluated PFS and OS on the basis of circulating biomarker changes from baseline to the first clinical evaluation. No significant differences were observed with respect to PFS. Patients in the B group with ≥30% reduction in eNOS levels showed a longer OS than those with <30% reduction (median OS 31.6 months, 95% CI 21.3–42.9 months *vs*.14.4 months, 95% CI 11.2–27.5 months, respectively; hazard ratio [HR 0.44], 95% CI 0.21–0.91, P = 0.027) (Table 2 and Fig. 1A,B). Patients in the same arm with >30% reduction in VEGF levels had a better OS than those with <30% reduction (median OS 28.8 months, 95% CI 17.8–42.9 months *vs*. 15.9 months, 95% CI 8.7–27.5, respectively; HR 0.52, 95% CI 0.27–1.02, P = 0.057) (Table 2 and Fig. 1C,D).

**Table 2:** PFS and OS with respect to changes in biomarker levels from baseline to first clinical evaluation.

Biomarker change	No. patients	PFS (months)				OS (months)			
		No. events	Median value (95% CI)	HR (95% CI)	P	No. events	Median value (95% CI)	HR (95% CI)	P
**CT** + **B**									
VEGF-A <30%	24	20	8.8 (6.1–12.5)	1.00		19	15.9 (8.7–27.5)	1.00	
≥30%	27	24	11.9 (9.1–15.7)	0.86 (0.47–1.58)	0.637	17	28.8 (17.8–42.9)	0.52 (0.27–1.02)	0.057
COX-2 <30%	11	8	18.7 (6.1–41.7)	1.00		6	41.7 (6.8–47.1)	1.00	
≥30%	40	36	9.9 (8.6–12.4)	2.00 (0.88–4.54)	0.097	30	21.3 (14.6–28.8)	1.57 (0.64–3.84)	0.319
HIF-1α <30%	21	17	9.1 (6.2–25.1)	1.00		16	20.1 (11.2–33.1)	1.00	
≥30%	30	27	11.4 (9.1–14.9)	1.11 (0.59–2.07)	0.752	20	25.2 (14.4–36.7)	0.93 (0.47–1.87)	0.849
EPHB4 <30%	21	17	9.1 (6.1–15.9)	1.00		16	19.3 (8.7–33.1)	1.00	
≥30%	30	27	10.8 (9.1–14.9)	1.01 (0.54–1.87)	0.983	20	25.2 (14.6–36.7)	0.64 (0.33–1.25)	0.189
eNOS <30%	33	28	9.1 (6.8–10.2)	1.00		24	14.4 (11.2–27.5)	1.00	
≥30%	18	16	14.1 (10.6–18.7)	0.66 (0.35–1.23)	0.191	12	31.6 (21.3–42.9)	0.44 (0.21–0.91)	0.027
**CT**									
VEGF-A <30%	30	27	9.1 (7.4–10.4)	1.00		22	23.2 (16.6–29.1)	1.00	
≥30%	27	25	11.4 (8.9–15.0)	0.76 (0.44–1.32)	0.326	19	26.4 (20.2–30.2)	0.83 (0.45–1.53)	0.545
COX-2 <30%	20	20	8.9 (6.3–9.5)	1.00		17	23.2 (16.6–28.0)	1.00	
≥ 30%	37	32	11.4 (9.0–15.0)	0.41 (0.22–0.75)	0.004	24	26.4 (20.2–36.7)	0.66 (0.35–1.23)	0.194
HIF-1α <30%	26	24	9.0 (6.5–9.3)	1.00		21	22.0 (14.4–28.6)	1.00	
≥30%	31	28	12.1 (9.0–16.2)	0.62 (0.36–1.08)	0.092	20	26.5 (20.4–30.2)	0.72 (0.39–1.34)	0.302
EPHB4 <30%	24	23	8.9 (6.5–9.5)	1.00		19	20.8 (16.6–27.1)	1.00	
≥30%	33	29	11.4 (9.0–16.2)	0.52 (0.29–0.92)	0.024	22	28.0 (20.4–30.2)	0.66 (0.36–1.23)	0.192
eNOS <30%	33	30	9.3 (8.9–13.0)	1.00		22	27.1 (18.8–36.6)	1.00	
≥30%	24	22	10.3 (6.0–12.1)	1.26 (0.72–2.19)	0.420	19	23.2 (15.0–29.2)	1.32 (0.71–2.44)	0.379

PFS, progression-free survival; OS, overall survival; CT, chemotherapy; B, bevacizumab; HR, hazard ratio; CI, confidence interval.

**Figure 2:**
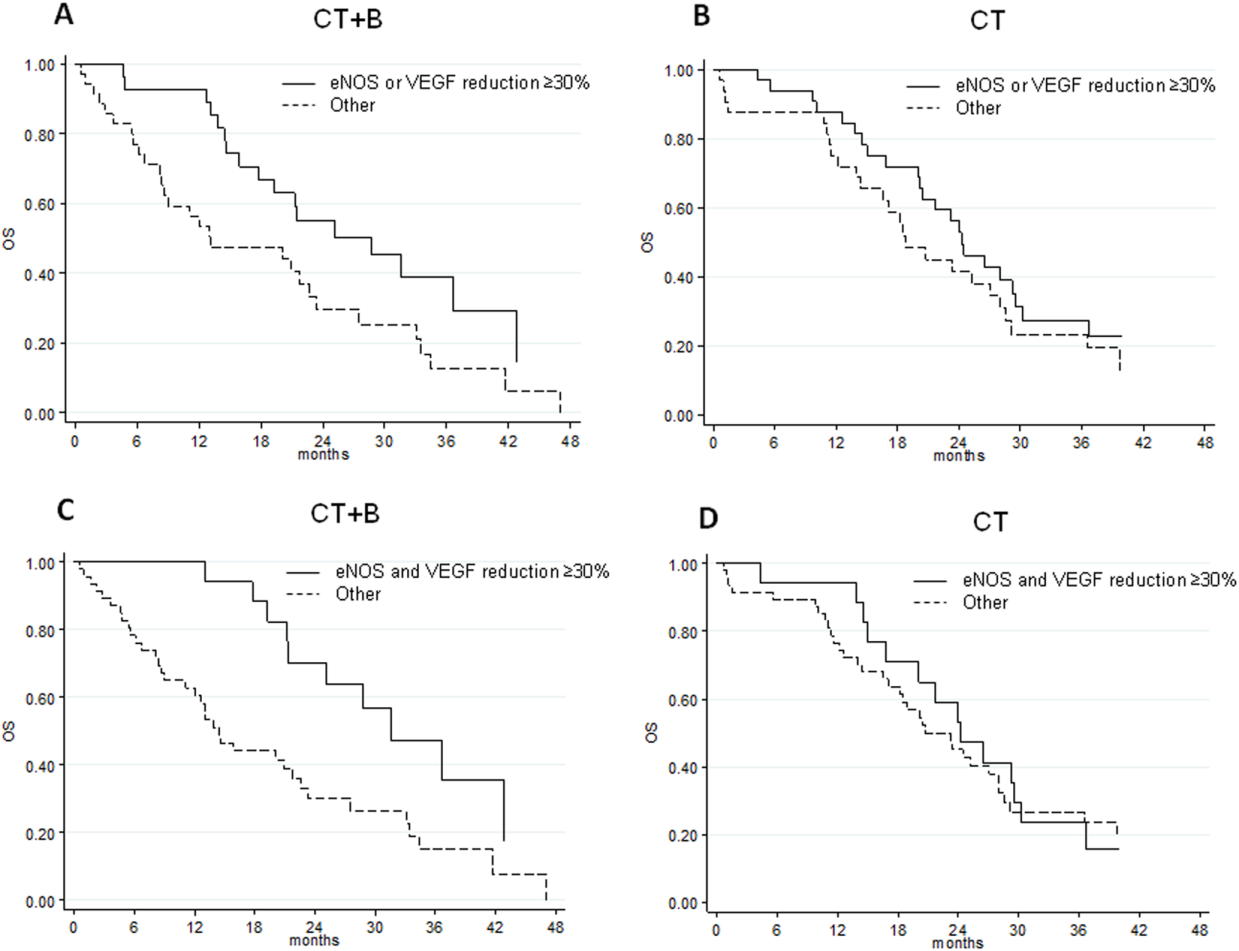
OS with respect to reduction in eNOS levels in CT + B (**A**) and CT (**B**) groups, and with respect to VEGF reduction in CT + B (**C**) and CT (**D**) groups.

Expression levels of VEGF and eNOS were significantly correlated in both CT + B- and CT groups (Spearman’s correlation coefficient = 0.80, P < 0.0001 *vs*. 0.75, P < 0.0001, respectively). However, in the B group, patients with >30% reduction in eNOS or VEGF levels showed a longer OS than the others without this reduction (median OS 28.8 months, 95% CI 15.9–42.9 *vs*. 13.1 months, 95% CI 8.2–22.7, respectively; HR 0.50, 95% CI 0.27–0.91, P = 0.023), (Table 3 and Fig. 2A,B).

**Table 3:** Reduction in eNOS and/or VEGF levels with respect to PFS/OS.

	No. patients	PFS (months)				OS (months)			
		No. events	Median PFS (95% CI)	HR (95% CI)	P	No. events	Median OS (95% CI)	HR (95% CI)	P
**CT + B**									
Other	23	19	8.1 (4.5–10.2)	1.00		18	13.1 (8.2–22.7)	1.00	
eNOS or VEGF reduction ≥ 30%	28	25	11.9 (9.2–15.9)	0.68 (0.40–1.16)	0.161	18	28.8 (15.9–42.9)	0.50 (0.27–0.91)	0.023
Other	34	29	9.0 (6.1–10.2)	1.00		25	14.4 (9.0–22.7)	1.00	
eNOS and VEGF reduction ≥30%	17	15	12.6 (9.2–22.3)	0.59 (0.32–1.07)	0.083	11	31.6 (21.3–49.5)	0.38 (0.19–0.78)	0.008
**CT**									
Other	24	21	9.1 (7.4–10.0)	1.00		17	18.8 (14.0–28.0)	1.00	
eNOS or VEGF reduction ≥30%	33	31	10.3 (7.2–12.2)	0.90 (0.54–1.50)	0.696	24	24.3 (20.0–29.6)	0.76 (0.43–1.34)	0.342
Other	39	36	9.0 (7.4–9.6)	1.00		27	20.8 (17.1–28.0)	1.00	
eNOS and VEGF reduction ≥30%	18	16	11.4 (8.9–15.0)	0.79 (0.45–1.41)	0.430	14	24.3 (15.0–30.2)	0.89 (0.48–1.65)	0.705

PFS, progression-free survival; OS, overall survival; HR, hazard ratio; CI, confidence interval; CT, chemotherapy; B, bevacizumab.

B-treated patients with >30% reduction in both eNOS and VEGF levels showed longer OS than other patients (median OS 31.6 months, 95% CI 21.3–49.5 *vs*. 14.4 months, 95% CI 9.0–22.7, respectively; HR 0.38, 95% CI 0.19–0.78, P = 0.008) (Table 3 and Fig. 2C,D). No significant differences were observed in terms of PFS. Furthermore, there were no substantial changes in biomarkers expression levels from baseline to progression (data not shown).

## Discussion

In this study we analyzed the circulating mRNA expression of 5 biomarkers, evaluated at baseline and during B treatment, to investigate their potential predictive role. We found that baseline circulating levels of biomarkers were not associated with clinical outcome, whereas >30% reduction in eNOS or VEGF levels from baseline to the first evaluation was associated with longer OS than in patients with <30% or no reduction. With regard to the 3 other biomarkers analyzed (HIF-1α, EPHB4 and COX-2), no significant correlations were seen between their variations during treatment and patient outcome.

Circulating markers offer a number of advantages over tissue-based markers, including the possibility of carrying out continuous and noninvasive assessments over time^19, 20^. VEGF is the most widely studied biomarker for predicting response to antiangiogenic treatment^21, 22^. An association between the efficacy of antiangiogenic treatments and VEGF tumor levels has been reported in several studies, with contrasting results^19, 23, 24^. VEGF plasma levels at baseline have shown prognostic value and have been correlated with metastatic potential and extension of colorectal cancer^5, 25, 26^. However, as VEGF values are dynamic, their change during treatment may be even more relevant than at baseline and could be used as a surrogate biomarker to predict response and progression^27^. This is in agreement with data published data by Gordon *et al*. who described a reduction in free serum VEGF levels in cancer patients treated with escalating doses of an anti-VEGF antibody compared to baseline serum concentrations^28^.

Similarly, Loupakis *et al*. showed that free VEGF levels measured after immunodepletion of plasma samples significantly decreased from baseline to day 14 among mCRC patients receiving B, suggesting that the anti-VEGF antibody effectively reduced the plasma level of the biologically active growth factor^29^. Conversely, other authors reported an increase in VEGF after treatment with B^8^. This discrepancy could be due to the different assays used and to the lack of discrimination between free and B-bound VEGF. We measured VEGF mRNA circulating levels and found that patients with a reduction in VEGF mRNA levels after B showed a better clinical outcome.

The novelty of our work lies in the measurement of eNOS levels at baseline and during treatment with a B-based therapy. As observed for VEGF levels, a >30% reduction in eNOS levels was associated with a better prognosis. Moreover, patients showing >30% reduction in both VEGF and eNOS levels showed longer survival, suggesting that the inhibition of both proteins indicates a better response to the antiangiogenic treatment. Interestingly, no significant association with survival was found in the CT-only group, reinforcing the predictive value of a reduction in biomarker levels in relation to B efficacy. Given that these two biomarkers were significantly correlated with each other and that their reduction was associated with better outcome, we can hypothesized that the VEGF-VEGFR-eNOS pathway may be involved in the response to B-based therapy.

In a previous work we demonstrated that a specific *eNOS* haplotype combination (defined as *eNOS* Haplo1/Haplo1 and *eNOS* Haplo 2/Haplo2) was associated with a favorable outcome in terms of ORR, PFS and OS in mCRC patients treated with B^11^. We also evaluated eNOS level changes with respect to different *eNOS* genotypes. A substantial percentage of patients carrying the *eNOS* haplotype combination responded to B-based therapy, a high number of these showing >30% reduction in eNOS levels. Conversely, fewer than half of the patients carrying other *eNOS* genotypes responded to treatment, and a small number of these also showed >30% reduction in eNOS levels (data not shown). These findings are suggestive of the potential role of eNOS pathway during B treatment.

Our study is somewhat limited by its small sample size and requires further validation in a prospective, independent and larger case series. However, the results were obtained on a prospectively enrolled patient population treated homogeneously in a randomized, prospective phase III multicenter study (*ITACa* trial) featuring two treatment arms: CT + B *vs*. CT only.

In conclusion, mCRC patients with a concomitant reduction in VEGF and eNOS biomarker levels showed a better outcome to treatment, indicating that these biomarkers might be useful to monitor B efficacy.

## Methods

### Patients and sample collection

**Figure 3:**
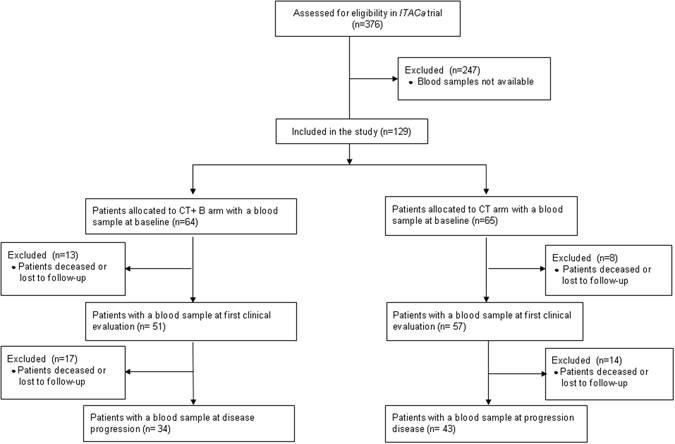
OS with respect to reduced eNOS or VEGF levels and reduced eNOS and VEGF levels in patients treated with CT + B ((**A**,**C**) respectively) or CT ((**B**,**D**) respectively).

This study included patients enrolled in the *ITACa* clinical trial^12^. Participation in the *ITACa* biological study was not mandatory for those taking part in the clinical trial. Of the 376 patients with mCRC enrolled in the *ITACa* trial, 129 had sufficient blood samples to be considered for this planned secondary analysis. Inclusion criteria, the randomization strategy and clinical results are described elsewhere^12^. Patients were randomized to receive first-line CT (FOLFOX4 or FOLFIRI) only or CT + B. CT + B doses and treatment details can be found in the original study article^12^. Sixty-four patients received CT + B and 65 patients received CT only (control group). Peripheral blood samples were collected in PAXgene tubes (PreAnalytix-Qiagen, Hilden, Germany) at various time points during the trial: at baseline (before the start of treatment), at the first evaluation (about 2 months later) and at disease progression (PD). Blood samples for 129 patients were available for analysis at baseline, for 108 (84%) at the first clinical evaluation and for 77 (60%) at PD (Fig. 3). Data were collected in accordance with good clinical practice.

All patients were evaluated for response, PFS and OS, in accordance with RECIST criteria version 1.1. Tumor response was assessed every 8 weeks by computed tomography. Responders included patients who obtained a complete response (CR) or partial response (PR). Non responders included those with stable disease (SD) or PD. The study was approved by the Local Ethics Committee (Ethics Committee Area Vasta Romagna and IRST) and informed consent for the use of biological material for research purposes was obtained from all patients before blood sample collection. The study was approved by the Local Ethics Committee (Ethics Committee Area Vasta Romagna and IRST) and informed consent for the use of biological material for research purposes was obtained from all patients before blood sample collection. All samples were collected in accordance with relevant guidelines and regulations.

### RNA extraction and amplification

Analyses of VEGF-A, COX2, HIF-1α, EPHB4 and eNOS were performed by biologists blinded to patient outcome. Total RNA was extracted by PAX-Gene blood RNA kit (PreAnalytix-Qiagen, Hilden, Germany) and RNA was treated with DNAse I. Five hundred nanograms of RNA were reverse-transcribed using the iScript cDNA Synthesis Kit (Bio-Rad, Hercules, CA, USA). The final reverse transcription mixture was incubated at 25 °C for 5 min, at 42 °C for 30 min and at 85 °C for 5 min. Real-time PCR was performed using the 7500 Applied Biosystems and TaqMan assay chemistry (Gene expression Assay, Applied Biosystems, Foster City, CA, USA). Two stably expressed endogenous β_2_-microglobulin (B2M) and hypoxanthine phosphoribosyltransferase 1 (HPRT1) genes were selected by Genorm software v. 3.2^30^ and were amplified and used as reference genes. All the RT-PCR experiments were run in duplicate.

### Statistical analysis

Gene expression analyses were performed by Applied Biosystems qPCR software. mRNA levels were normalized to endogenous reference B2M and HPRT genes. We used a healthy donor as calibrator. Relative quantification was calculated by the 2-delta delta Ct method. The aims of this planned secondary analysis were to examine the association between baseline circulating mRNA expression of VEGF-A, eNOS, EPHB4, COX2 and HIF-1α and PFS and OS in the *ITACa* population, and to investigate their variation during treatment in order to monitor B efficacy. We chose the median value of variation in the case series (30%) as the cut-off.

The primary aim of the *ITACa* study was PFS. Secondary efficacy endpoints were ORR and OS. PFS was calculated as the time from the date of randomization to the date of the first observation of PD (per investigator assessment), last tumor evaluation or death in the absence of progressive disease. Patients undergoing curative metastasectomy were censored at the time of surgery. OS was calculated as the time from the date of randomization to the date of death from any cause or last follow-up.

Descriptive statistics were used to describe enrolled patients. The relationship between baseline mRNA expression of VEGF-A, eNOS, EPHB4, COX2 and HIF-1α and clinical-pathologic factors was analyzed using a nonparametric ranking statistic (Median test). Spearman’s correlation coefficient was used to investigate the relationship between the mRNA levels considered as continuous variables.

Time to event data (PFS, OS) were described using the Kaplan-Meier method and compared using the log rank test (at a significance level of 5%). 95% CIs were calculated by nonparametric methods. Estimated HRs and their 95% CI were calculated by the Cox regression model. We also conducted landmark analysis to reduce the potential for time-dependent confounding in treatment by assessing the impact of changes in mRNA levels from baseline to the first tumor evaluation (about 2 months after the start of treatment) on survival outcome. Patients who were still alive and had not been lost to follow-up at the landmark time were divided into two categories on the basis of whether they had progressed or not by that time. PFS and OS after the landmark time were computed with Kaplan-Meier curves.

The correlation between circulating mRNA expression of VEGF-A, eNOS, EPHB4, COX2 and HIF-1α and clinical outcome was analyzed separately in each treatment group (CT + B and CT). All P-values were based on two-sided testing and statistical analyses were carried out using SAS statistical software version 9.4 (SAS Institute, Cary, NC, USA).

## Electronic supplementary material

Supplementary Tables S1-S3
