# Supplementary material for: Circulating VEGF and eNOS variations as predictors of outcome in metastatic colorectal cancer patients receiving bevacizumab
Source: Sci Rep. 2017 May 2;7:1293. doi: 10.1038/s41598-017-01420-0 (PMC5431064; doi:10.1038/s41598-017-01420-0)
Supplement: Supplementary file 1 — Supplementary Tables S1-S3 [file 41598_2017_1420_MOESM1_ESM.doc]

**Circulating *VEGF* and *eNOS* variations as predictors of outcome in metastatic colorectal cancer patients receiving bevacizumab**

Giorgia Marisi, Emanuela Scarpi, Alessandro Passardi, Oriana Nanni, Angela Ragazzini, Martina Valgiusti, Andrea Casadei Gardini, Luca Maria Neri, Giovanni Luca Frassineti, Dino Amadori & Paola Ulivi

**Supplementary Table S1. Correlation between median biomarker values and clinical-pathological features**

|  | **VEGF** | **COX** | **HIF1** | **EPHB** | **eNOS** |
| --- | --- | --- | --- | --- | --- |
|  | **Median value (range)** | | | | |
| **Gender** |  |  |  |  |  |
| Male | 2.33 (0.72-34.87) | 1.20 (0.37-6.07) | 1.17 (0.35-5.38) | 2.92 (0.35-78.20) | 6.80 (0.53-118.15) |
| Female | 2.30 (0.54-50.80) | 1.25 (0.34-4.78) | 1.06 (0.28-4.23) | 3.36 (0.21-129.51) | 5.86 (0.41-123.16) |
|  |  |  |  |  |  |
| **Performance Status** (ECOG) |  |  |  |  |  |
| 0 | 2.29 (0.68-50.80) | 1.26 (0.34-6.07) | 1.11 (0.28-5.38) | 3.02 (0.37-129.51) | 6.52 (0.41-123.16) |
| 1-2 | 2.50 (0.54-19.94) | 1.17 (0.37-2.40) | 1.37 (0.43-3.38) | 4.08 (0.21-39.80) | 7.48 (0.59-85.43) |
|  |  |  |  |  |  |
| **Tumor localization** |  |  |  |  |  |
| Rectum | 2.59 (0.72-37.69) | 1.37 (0.34-4.54) | 1.41 (0.41-4.23) | 4.16 (0.38-64.80) | 7.67 (0.59-123.16) |
| Colon | 2.22 (0.54-50.80) | 1.20 (0.37-6.07) | 1.07 (0.28-5.38) | 2.87 (0.21-129.51) | 6.61 (0.41-118.15) |
|  |  |  |  |  |  |
| Right side | 2.27 (0.54-50.80) | 1.13 (0.37-4.78) | 1.08 (0.34-5.38) | 2.70 (0.21-129.51) | 4.88 (0.41-118.15) |
| Left side | 2.36 (0.68-37.69) | 1.37 (0.34-6.07) | 1.17 (0.28-4.23) | 3.57 (0.38-69.55) | 7.14 (0.59-123.16) |
|  |  |  |  |  |  |
| **Stage at diagnosis** |  |  |  |  |  |
| I-III | 2.75 (0.54-50.80) | 1.32 (0.39-4.78) | 1.30 (0.35-3.94) | 3.02 (0.21-129.51) | 7.67 (0.88-67.14) |
| IV | 2.24 (0.72-37.69) | 1.23 (0.34-6.07) | 1.11 (0.28-5.38) | 3.10 (0.35-78.20) | 6.62 (0.41-123.16) |
|  |  |  |  |  |  |
| **Grade** |  |  |  |  |  |
| 1+2 | 2.37 (0.68-37.69) | 1.19 (0.34-4.54) | 1.09 (0.28-4.23) | 2.87 (0.35-69.55) | 7.41 (0.53-123.16) |
| 3 | 2.30 (0.54-50.80) | 1.25 (0.42-6.07) | 1.27 (0.34-5.38) | 3.01 (0.21-129.51) | 5.22 (0.41-118.15) |
|  |  |  |  |  |  |
| ***KRAS* status** |  |  |  |  |  |
| Wild type | 2.23 (0.68-37.69) | 1.26 (0.34-6.07) | 1.10 (0.28-5.38) | 3.14 (0.35-64.80) | 6.50 (0.49-57.34) |
| Mutated | 2.75 (0.54-50.80) | 1.31 (0.39-4.78) | 1.38 (0.35-4.01) | 3.33 (0.21-129.51) | 6.83 (0.41-123.16) |
|  |  |  |  |  |  |

**Supplementary Table S2. Median biomarker values at baseline in relation to objective response**

| **Median biomarker value**  **(range, IQR) at baseline** | **Responders (CR+PR)**  **(n=37)** | **Non responders (SD+PD)**  **(n=25)** | **P** |
| --- | --- | --- | --- |
| **CT + B** |  |  |  |
| VEGF-A | 2.38 (0.54-50.80, 2.40) | 2.55 (0.68-21.47, 4.99) | 0.714 |
| COX-2 | 1.27 (0.34-4.78, 0.94) | 0.97 (0.24-4.09, 0.96) | 0.121 |
| HIF-1α | 1.17 (0.28-5.38, 0.85) | 1.08 (0.34-4.01, 1.92) | 0.315 |
| EPHB4 | 3.10 (0.21-129.51, 4.39) | 2.65 (0.37-69.55, 7.67) | 0.754 |
| eNOS | 6.15 (0.41-67.14, 6.27) | 5.67 (0.85-65.49, 18.93) | 0.957 |
|  | **Responders**  **(n=38)** | **Non responders**  **(n=27)** | **P** |
| **CT** |  |  |  |
| VEGF-A | 2.36 (0.49-34.87, 1.89) | 2.22 (0.96-23.93, 5.76) | 0.438 |
| COX-2 | 1.34 (0.37-4.20, 0.78) | 1.44 (0.45-6.07, 1.09) | 0.482 |
| HIF-1α | 1.12 (0.27-3.17, 0.80) | 1.30 (0.53-3.94, 1.61) | 0.113 |
| EPHB4 | 3.28 (0.35-78.20, 3.22) | 3.14 (0.56-47.83, 14.43) | 0.709 |
| eNOS | 7.25 (1.14-118.15, 7.97) | 6.52 (0.49-123.16, 14.15) | 0.875 |

CT, chemotherapy; B, bevacizumab; CR: complete response; PR: partial response; SD: stable disease; PD: progressive disease

**Supplementary Table S3. PFS and OS in relation to median baseline biomarker values**

|  | **CT+B (n=64)** | | | | | **CT (n=65)** | | | | |
| --- | --- | --- | --- | --- | --- | --- | --- | --- | --- | --- |
| **Median baseline value** | **No. patients** | **Median PFS (months)**  **(95% CI)** | **P** | **Median OS (months)**  **(95% CI)** | **P** | **No. patients** | **Median PFS (months)**  **(95% CI)** | **P** | **Median OS (months)**  **(95% CI)** | **P** |
| **VEGF-A** ≤2.32 | 34 | 9.1 (6.8-12.4) |  | 20.1 (13.1-31.6) |  | 31 | 8.9 (6.5-9.5) |  | 20.8 (17.1-37.3) |  |
| >2.32 | 30 | 9.4 (6.9-13.1) | 0.550 | 21.8 (12.7-33.1) | 0.688 | 34 | 10.0 (8.9-12.1) | 0.390 | 24.3 (14.5-28.0) | 0.282 |
| **COX-2** ≤1.23 | 31 | 9.2 (6.8-14.9) |  | 25.2 (13.1-35.7) |  | 35 | 8.3 (6.3-9.1) |  | 20.8 (15.0-28.0) |  |
| >1.23 | 33 | 9.0 (6.9-10.6) | 0.307 | 20.1 (12.7-27.1) | 0.502 | 30 | 10.0 (8.9-21.5) | 0.008 | 24.5 (17.1-30.2) | 0.273 |
| **HIF-1α** ≤1.12 | 31 | 9.4 (8.1-12.9) |  | 23.4 (13.1-33.5) |  | 35 | 8.9 (6.5-9.1) |  | 20.8 (16.6-29.6) |  |
| >1.12 | 33 | 9.0 (6.8-10.9) | 0.788 | 21.3 (9.0-27.1) | 0.895 | 30 | 10.3 (8.3-15.0) | 0.065 | 24.3 (16.8-29.1) | 0.813 |
| **EPHB4** ≤3.04 | 31 | 10.2 (8.3-14.7) |  | 22.7 (13.9-35.7) |  | 35 | 8.3 (6.5-9.1) |  | 23.2 (16.6-28.6) |  |
| >3.04 | 33 | 8.6 (6.8-10.6) | 0.423 | 21.3 (9.0-27.1) | 0.462 | 30 | 10.3 (8.9-13.1) | 0.100 | 24.5 (16.8-29.2) | 0.788 |
| **eNOS** ≤6.52 | 31 | 8.9 (6.1-10.2) |  | 14.6 (8.7-27.5) |  | 34 | 8.9 (6.5-9.5) |  | 23.7 (16.6-29.6) |  |
| >6.52 | 33 | 9.7 (7.7-13.1) | 0.228 | 23.4 (15.9-33.5) | 0.382 | 31 | 9.6 (7.8-12.1) | 0.691 | 24.0 (16.8-29.1) | 0.796 |

CT, chemotherapy; B, bevacizumab; PFS, progression-free survival; OS, overall survival
